# Foundation and Clinical Evaluation of a New Method for Detecting SARS-CoV-2 Antigen by Fluorescent Microsphere Immunochromatography

**DOI:** 10.3389/fcimb.2020.553837

**Published:** 2020-11-30

**Authors:** Chunyan Zhang, Lei Zhou, Kang Du, Ying Zhang, Jing Wang, Lijuan Chen, Yanning Lyu, Jun Li, Hao Liu, Junli Huo, Fei Li, Jiayi Wang, Peipei Sang, Si Lin, Yi Xiao, Kan Zhang, Kunlun He

**Affiliations:** ^1^ Birth Defects Prevention and Control Technology Research Center, Chinese PLA General Hospital, Beijing, China; ^2^ Clinical Laboratory, Wuhan Huoshenshan Hospital, Wuhan, China; ^3^ Clinical Laboratory, Xijing Hospital of Air Force Medical University of PLA, Xi’an, China; ^4^ School of Precision Instruments and Optoelectronics Engineering, Tianjin University, Tianjin, China; ^5^ Clinical Laboratory, First Medical Center of Chinese PLA General Hospital, Beijing, China; ^6^ Clinical Laboratory, Chongqing Public Health Medical Center, Southwest University Public Health Hospital, Chongqing, China; ^7^ Institute of Infectious and Endemic Diseases Prevention and Control, Beijing Center for Diseases Prevention and Control, Beijing, China; ^8^ Infections Department, Wuhan Huoshenshan Hospital, Wuhan, China; ^9^ Neurosurgery Department, Xijing Hospital of Air Force Medical University of PLA, Xi’an, China; ^10^ Medical Department, Wuhan Huoshenshan Hospital, Wuhan, China; ^11^ Cardiovascular Medicine Department, Xijing Hospital of Air Force Medical University of PLA, Xi’an, China; ^12^ Beijing Savant Biotechnology Co., Ltd., Beijing, China; ^13^ Translational Medicine Research Center, Key Laboratory of Ministry of Industry and Information Technology of Biomedical Engineering and Translational Medicine, Chinese PLA General Hospital, Beijing, China

**Keywords:** SARS-CoV-2, COVID-19, clinical evaluation, antigen detection, immunochromatographic

## Abstract

**Purpose:**

To develop a rapid detection reagent for SARS-CoV-2 antigen for the auxiliary diagnosis of new coronary pneumonia (COVID-19), and perform the methodological evaluation and clinical evaluation of the reagent.

**Method:**

SARS-CoV-2 N-protein test strip was created by combining fluorescent microsphere labeling technology and immunochromatographic technology, based on the principle of double antibody sandwich. Then we evaluated the analytical capability and clinical application of the strips.

**Result:**

The limit of detection of the strips for recombinant N protein was 100 ng/ml and for activated SARS -CoV-2 virus was 1 × 10^3^ TCID_50_/ml. The strips also have high analytical specificity and anti-interference capability. According to the predetermined cut-off value, the specificity of the test strip in healthy controls and patients with other respiratory disease was 100.00 and 97.29%, the sensitivity in COVID-19 cases at progress stage and cured stage was 67.15 and 7.02%. The positive percentage agreement and negative percentage agreement of antigen strip to RNA test were 83.16 and 94.45%.

**Conclusion:**

SARS-CoV-2 fluorescence immunochromatographic test strip can achieve fast, sensitive and accurate detection, which can meet the clinical requirements for rapid detection of viruses on the spot.

## Introduction

In December 2019, a number of cases of viral pneumonia were found in Wuhan, China, and the initial cases were related to the exposure in Wuhan seafood market (Jiang et al., 2020; Wu et al., 2020). On February 11, 2020, the International Committee on Taxonomy of Viruses (ICTV) announced the official name of the new coronavirus: severe acute respiratory syndrome coronavirus 2 (SARS-CoV-2); simultaneously, the World Health Organization (WHO) announced that the new coronavirus-infected pneumonia was officially named “COVID-19” (Munster et al., 2020; Zhu N. et al., 2020). The COVID-19 has spread to 206 countries and regions in the world by April 4th, 2020, accounting for 88.4% of the total. More than 1 million cases of covid-19 have been confirmed worldwide, and more than 50,000 cases have died.

Coronavirus is a common positive-strand RNA virus that causes respiratory diseases, existing widely in nature. Humans, vertebrates and invertebrates can be their parasitic host (Guo et al., 2020). So far, 7 kinds of infectious coronavirus have been found, i.e. HCoV-229-E, HCoV-OC43, SARS-CoV, HCoV-NL63, HCoV-HKU1, MERS-CoV and the recently discovered SARS-CoV-2 in Wuhan. Among those, 229E, NL63, OC43 and HKU1 can cause common cold symptoms, and the remaining three are highly pathogenic SARS-CoV (atypical pneumonia), MERS-CoV (Middle Eastern Respiratory Syndrome Coronavirus), and the newly discovered SARS-CoV-2.

According to the analysis of the SARS-CoV-2 database officially released by the National Genomics Science Data Center on January 22, the whole genome sequence of SARS-CoV-2 is 29903nt, which mainly includes the genes ORF1a and 1b encoding non-structural proteins and S, E, M, N encoding structural proteins. The M and E protein play a critical role in coordinating virus assembly and forming mature viral envelopes, while the N protein binds to the viral RNA and is involved in the transcription and replication of viral RNA, as well as packaging of the encapsidated genome into virions (Ashour et al., 2020; Nishiga et al., 2020; Zhu C. et al., 2020). Research shows that both SARS-CoV-2 in 2019 and the SARS outbreak in 2003 probably originate from the bat, and genome sequence similarity is up to 80%. Moreover, SARS-CoV-2 infection path and the pathogenesis are similar to SARS (Sun et al., 2020; Zhou et al., 2020), namely, the S-protein of SARS-CoV-2 and Angiotensin converting enzyme gene 2 (Angiotensin-converting enzyme 2, ACE2) interacting invades into the host cell, and then complete the replication of the virus (Prompetchara et al., 2020).

Pathogens are usually tested by molecular diagnosis and immunodiagnosis. The N protein can be exposed in the process of virus assembling, which make it become one of the targets of clinical detection. An article published reported detect the N protein of MERS-CoV with antigen detection method was feasible (Chen et al., 2015). In the early stage of the SARS-CoV-2 outbreak, the fluorescence PCR method was adopted in preference. The results of this method were accurate, but there were also some problems such as complicated operation and susceptibility to environmental factors. Therefore, the rapid immunodiagnostic reagents can be used for screening in the middle and late stages of the epidemic prevention. In the process of developing immunoassay reagents, the specific and conserved sequence of viral N protein was selected through the published genome sequences, a large number of antibodies were screened, and then the viral antigens were detected by the method of double-antibody sandwiched antigens.

## Materials and Methods

### Patients and Samples

Nasal/oropharyngeal swabs of a total of 990 samples from January 2020 to April 2020 were collected and tested in this study, including 247 COVID-19 patients, 443 patients with other respiratory diseases and 300 healthy people. Nasal/oropharyngeal swab samples were collected from the patients/healthy people according to standard operation, stored and transported within a single tube of Hanks virus preservation solution (ph7.4–7.6) (Beijing Youkang Technology Co., Ltd) to prevent viral RNA/protein degradation.

### Reagents and Equipment

Mouse anti-SARS-CoV-2 N protein monoclonal antibody-1 and mouse anti-SARS-CoV-2 N protein monoclonal antibody-2 were purchased from Beijing Biosynthesis Biotechnology Co., Ltd.; The recombinant N protein of SARS-CoV-2 were donated by Tianjin University; The rabbit IgG was purchased from Beijing Mingchaoxi Technology Co., Ltd. Company; The sheep anti-rabbit secondary antibody was purchased from Kema Biotechnology (Beijing) Co., Ltd.; The latex fluorescent microspheres were purchased from Ocean Nanotech (USA); The nitrocellulose membrane was purchased from Merck Chemical Technology (Shanghai) Co., Ltd.; The glass cellulose membrane was purchased from Shanghai Joey Biotechnology Co., Ltd.; The PVP bottom plate was purchased from Shanghai Kinbio Biotechnology Co., Ltd.; The N-hydroxysuccinimide (NHS), Carbonized (EDC) and the biological preservatives were purchased from Sigma-Aldrich (Shanghai) Trading Co., Ltd. The Savant-100 fluorescent immunochromatography analyzer was purchased from Beijing Savant Biotechnology Co., Ltd. The Symphony-100 fluorescence immunoanalyzer was purchased from Tianjin Boomscience Technology Co., Ltd. Beijing Savant Biotechnology Co., Ltd. is responsible for the small batch production of SARS-CoV-2 antigen test strips and packaging materials.

### Method

**Figure 1:**
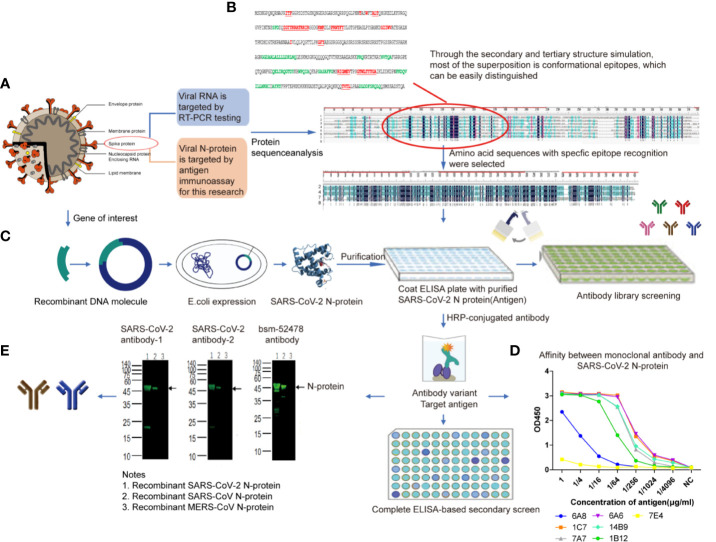
SARS-CoV-2 N-protein specific antibody selection and cross reaction **(A)**. SARS-CoV-2 N-protein was chose for antigen immunoassay **(B)**. The sequence comparison between SARS-CoV-2 N-protein and that of the other 6 kinds of coronaviruses. From the top to the bottom of the list: 1, HCoV-229E (AOG74787.1_N); 2, SARS-CoV-2 (YP009724397.2_N); 3, HCoV-HKU1(AGW27885.1_N); 4,MERS-CoV (AHC74105.1_N); 5, HCoV-NL63 (AFV53152.1_N); 6, HCoV-OC43 (QDH43730.1_N); 7, SARS-CoV ShanghaiQXC1(AAR86795.1_N); 8,consensus sequence, the black area **(C)**. The expression of SARS-CoV-2 N-protein **(D)**. Affinity determination between monoclonal antibody and SARS-CoV-2 N-protein. 6A8, 6A6, 7e4, 1c7, 14b9, 7a7, and 1B12 represent seven monoclonal antibodies selected from antibody library of Beijing Biosynthesis Biotechnology Co. Ltd **(E)**. Cross-reaction result. Anti-SARS-CoV-2 N-protein monoclonal antibody-1 and antibody-2 were selected as immune target.

**Figure 2:**
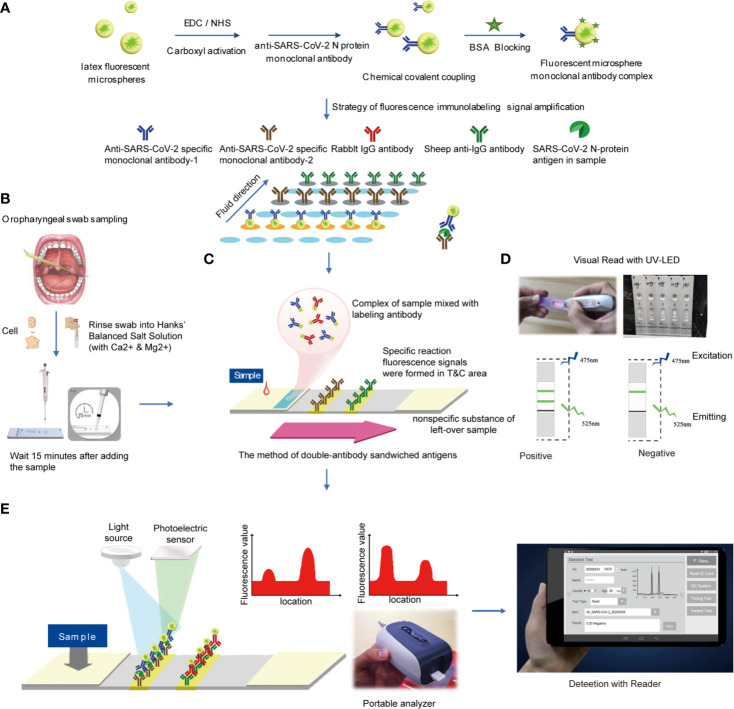
Reagent preparation and detection process **(A)**. Preparation of antibody detection reagent strip **(B)**. Oropharyngeal swab sampling **(C)**. The method of double-antibody sandwiched antigens **(D)**. Read the result using UV-LED **(E)**. Analyzed by the detector.

The design and procedure are illustrated schematically in **Figures 1** and **2**.

(1) Recombinant SARS-CoV-2 N protein

According to the sequence of SARS-CoV-2 (Wuhan, Accession: QHD43423.2), the gene of N protein was synthesized and inserted into pET28a vector. The recombinant SARS-CoV-2 N protein was obtained by inducing expression in E. coli and purified by Ni affinity column.

(2) Preparation of test strips

Firstly, 1 ml of latex fluorescent microspheres, 1 mg EDC and 1 mg NHS were mixed and stirred at room temperature for 3 h. Then 0.1 mg mouse anti-SARS-CoV-2 N protein monoclonal antibody-2 was added and stirred at room temperature for 1 h. Following that, 10 mg BSA blocking solution was added and stirred for 1 h. The centrifugation was performed at 2–8°C for 30 min at 11,000 r/min to remove the supernatant. Finally, the solid precipitate was redissolved to 1 ml of phosphate buffer solution (1 mol/L, pH = 7.4), 1 μL of Proclin 300 was added, and the mixture was stored at 4°C for use. In the same way, the rabbit IgG was labeled with latex fluorescent microspheres and stored at 4°C for use.

(3) Preparation of coating pads

The mouse anti-SARS-CoV-2 N protein monoclonal antibody-1 and the sheep anti-rabbit secondary antibody were diluted with phosphate buffer solution, respectively. The above solution was coated on the NC membrane at a concentration of 1 mg/ml, using a gold film sprayer. The line with mouse anti-SARS-CoV-2 N protein monoclonal antibody-1 was set as the test line (T line), and the other with sheep anti-rabbit secondary antibody was set as the quality control line (C line). The as-prepared pads were dried at 37°C with humidity <30% for 4 h.

(4) Preparation of marker pads

The latex fluorescent microsphere-labeled mouse anti-SARS-CoV-2 N protein monoclonal antibody-2 and latex fluorescent microsphere-labeled rabbit IgG were mixed in a volume ratio of 1:1 and sprayed on glass cellulose membrane at a rate of 10 μL/cm. Then the marker pad was dried at 45°C for 4 h.

(5) Test strip assembly

First, the coating pad was pasted on the PVC base. Then the absorbent pad near the C line on the NC film and the marker pad on the end near the T line were connected and cut into 4 ± 0.1 mm test strip with a slitter.

### Detection

Antigen detection method with test strips was described as follows (1): Take out the test card at room temperature, cut the package and lay it on the table for later use (2). Take out the standard card in the kit, read the curve information in the standard card at the IC card sensing position of the fluorescence immunochromatograph and store it in the analyzer (3); Before testing, select the curve information consistent with the item and batch number (4); Add 60 μL of sample solution to each test strip, leave it at room temperature for 15 min and then insert it into the fluorescence immunochromatography analyzer for detection. The instrument will automatically calculate the sample concentration value (**Figure 2E**).

Detection of SARS-CoV-2 nucleic acid RNA was performed by fluorescence quantitative PCR: nucleic acid extraction was operated according to the literature (Mollica, 2010), and the fluorescence quantitative PCR process was operated according to the instructions.

### Data Analysis and Statistics

Statistics analysis was performed with the software SPSS 20.0, and nonparametric test and two-side χ2 test were used to compare the differences between the two groups. A receiver operating characteristic (ROC) analysis was constructed to determine the best cut-off value to predict the outcome. The probability was calculated using a logistic regression model, and the estimated probabilities were used in a ROC analysis to calculate the area under curve (AUC) for different models. P value < 0.05 will be considered statistically significant.

## Results

### Limit of Detection (LoD) of Recombinant N Protein

SARS-CoV-2 recombinant N protein (concentration: 0.5 mg/ml) was used to prepare samples at concentration of 50, 100, 200, 500, and 1 μg/ml. We detected samples at each concentration 20 times by test strips at three batches, and defined the lowest concentration with positive results over 19/20 replicates as the limit of detection. As shown in **Table 1**, 100 ng/ml was defined as the limit of detection for the strips.

**Table 1:** Limit of detection.

	50 ng/ml*			100 ng/ml*			750 TCID_50_/ml**			1000 TCID_50_/ml**		
	Lot 1	Lot 2	Lot 3	Lot 1	Lot 2	Lot 3	Lot 1	Lot 2	Lot 3	Lot 1	Lot 2	Lot 3
No. tested	20	20	20	20	20	20	20	20	20	20	20	20
Positive results	18	18	16	20	20	20	15	20	16	20	20	20
Positive ratio	88.33%			100%			85.00%			100%		

*tests used with recombinant protein-N of SARS-CoV-2; **tests used with culture fluid of SARS-CoV-2. The results were detected by a Savant-100 Fluorescence immunochromatographic analyzer.

### LoD of Activated SARS -CoV-2 Virus

The LoD for the activated SARS-CoV-2 virus (IVCAS 6.7512, Wuhan Institute of Virology. CSA.) was tested, in both the sample preservation solution and real clinical matrix (sample preservation solution mixed with orophyngeal swabs samples of healthy people). The experiments were conducted in the P3 Biosafety Protection Laboratory. Initially, the activated SARS-CoV-2 culture medium (host cell: Vero E6 cell line, ATCC CRL-1586 ™) was diluted at gradients of 1:1, 1:2, 1:20, 1:400 and 1: 2,000, each sample was tested at least twice, and the dilution at 1:2,000 was tested for 13 times. Then, the concentration of 1:2,000 (100% positive,13/13) was used as the LoD range to expand the testing for the LoD. Dilutions with concentrations of 2 × 10^3^, 1 × 10^3^, and 7.5 × 10^2^ TCID_50_/ml were tested for 20 times, respectively. These results revealed a 100% (20/20) positive result at 1×10^3^ TCID_50_/ml concentration. Hence, 1 × 10^3^ TCID_50_/ml was set as the LoD (**Table 1**).

### Anti-Interference

The experiment investigated the interference to the test results of common interference substances in samples such as mucin, blood, nasal cavity drugs, antiviral drugs, antibiotics, and so on. The samples of the same antigen concentration were mixed with different interfering substances, and then the strips in same batch were used for the test. As shown in **Table 2**, the test results had no deviation from the detection results of the SARS-CoV-2, which indicated that the common interfering substances in the samples did not interfere with the experimental results.

**Table 2:** Concentration table of interfering substances.

Substances	Concentration	Result
Full blood	2.2%	Negative
Mucin	250 μg/mL	Negative
Guaifenesin	20 mg/ml	Negative
Ribavirin	10 mg/ml	Negative
(R)-(-)-Phenylephrine hydrochloride	200 mg/ml	Negative
Chlorpheniramine Maleate	25 mg/ml	Negative
Levofloxacin	20 mg/ml	Negative
Tobramycin	5 mg/ml	Negative
Lopinavir	20 mg/ml	Negative
Oseltamivir phosphate Ritonavir Peramivir Trihydrate Cephradine Zanamivir Flunisolide Fluticasone propionate Dexamethasone Mometasone Furoate Beclomethasone Triamcinolone acetonide Fluocinolone Acetonide Azithromycin Meropenem trihydrate Ceftriaxone Sodium Trihydrate Budesonide Arbidol hydrochloride HUMAN IFN-ALPHA A	20 mg/ml 2.5 mg/ml 0.2 mg/ml 20 mg/ml 20 mg/ml 5 mg/ml 15 mg/ml 10 mg/ml 10 mg/ml 2 mg/ml 2 mg/ml 10 mg/ml 20 mg/ml 5 mg/ml 25 mg/ml 40 mg/ml 10 mg/ml 0.2 mg/ml	Negative Negative Negative Negative Negative Negative Negative Negative Negative Negative Negative Negative Negative Negative Negative Negative Negative Negative

### Cross-Reaction

Affinity experiment between monoclonal antibody (From antibody library of Beijing Biosynthesis Biotechnology Co. Ltd.) and SARS-CoV-2 N protein showed the optimum working concentration of selected antibody (**Figure 1D**). Western blotting (WB) (Algenas et al., 2014) cross-reaction experiments were conducted between two monoclonal antibodies of SARS-CoV-2 recombinant N protein and recombinant N protein of SARS-CoV and MERS-CoV, in order to verify the specificity of antibodies (**Figure 1E**). The results of cross-reaction detection showed that the antibody had cross-reactivity towards recombinant N protein of SARS-CoV and no cross-reactivity towards that of other 7 kinds of coronavirus (**Table 3**), which was consistent with other studies (Saahir Khan et al., 2020). For the SARS-CoV N protein which produced a positive response, we diluted the sample into concentrations of 1, 500, 200, 100, and 50 ng/ml. The results showed when SARS-CoV N protein was diluted to concentration of 50 ng/ml, there was no cross-reactivity (**Table 3**).

**Table 3:** Results of different pathogen samples.

Item	Substance	Source	Concentration	Test Result
1	Dilution	Hanks’ balanced salt solution	–	Negative
2	HCoV-HKU1 N protein	Genetically engineered virus recombinant N protein	1 μg/mL	Negative
3	HCoV-OC43 N protein	Genetically engineered virus recombinant N protein	1 μg/mL	Negative
4	HCoV-229E N protein	Genetically engineered virus recombinant N protein	1 μg/mL	Negative
5	HCoV-NL63	Genetically engineered virus recombinant N protein	1 μg/mL	Negative
6	HKU8 N protein (from bat)	Genetically engineered virus recombinant N protein	1 μg/mL	Negative
7	HKU10 N protein (from bat)	Genetically engineered virus recombinant N protein	1 μg/mL	Negative
8	MERS-CoV	Genetically engineered virus recombinant N protein	1 μg/mL	Negative
9	SARS-CoV N protein	Genetically engineered virus recombinant N protein	1 μg/mL	Positive
	SARS-CoV N protein	Genetically engineered virus recombinant N protein	500 ng/ml	Positive
	SARS-CoV N protein	Genetically engineered virus recombinant N protein	200 ng/ml	Positive
	SARS-CoV N protein	Genetically engineered virus recombinant N protein	100 ng/ml	Positive
	SARS-CoV N protein	Genetically engineered virus recombinant N protein	50 ng/ml	Negative

### Precision Verification

According to the requirements of EP15-A3, the negative(N1\N2), weakly positive (S1\S2) and strong positive (P1\P2) samples were tested 5 times a day for 5 days, and the total precision of all samples was calculated for positive or negative percentage agreement. The results in **Table 4** showed that except the positive percentage agreement of S2 samples was 96%, the percentage agreements of other samples were all 100%. All antigen kits in test showed uniform fluorescence distribution under UV-LED excitation.

**Table 4:** Precision.

	Negative		Weakly Positive		Strong Positive	
Sample	N1	N2	S1	S2	P1	P2
NPA*	100%	100%	/	/	/	/
PPA*	/	/	100%	96%	100%	100%

NPA respond to Negative Percentage Agreement, PPA respond to Positive Percentage Agreement.

### Cutoff Value Determination

**Figure 3:**
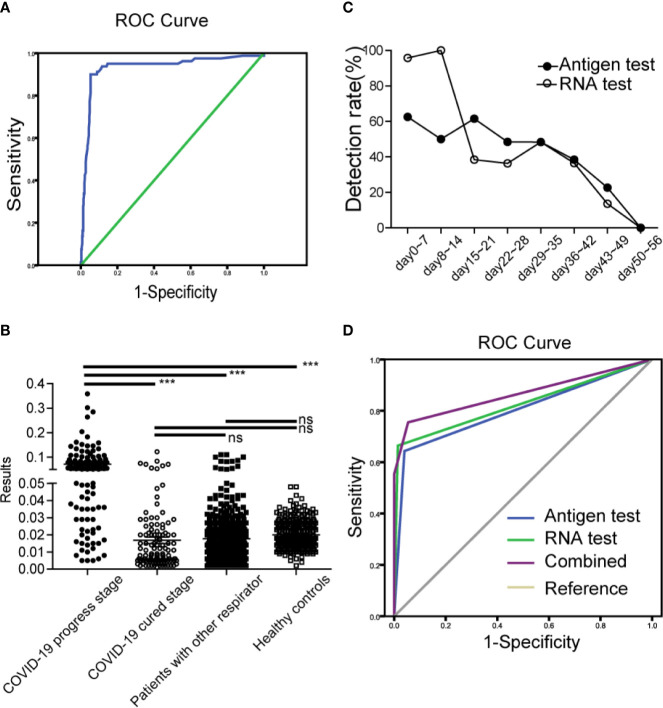
Clinical application and evaluation of antigen strips **(A)**. ROC curve for the cutoff determine of antigen strips **(B)**. The results of antigen strips in COVID-19 cases (progress stage, ●; cured stage, ○), patients with other respiratory diseases (■), and healthy controls (□). ***p < 0.001; ns, nonsense **(C)**. The antigen detection rate at different time of development of COVID-19. Antigen test (●), RNA test (○) **(D)**. Diagnosis value of antigen strips, RNA detection and combined evaluation.

To investigate how sensitive and specificity the assay is, in our primary experiment, 306 samples (age range 2–86, average 40.12 ± 14.33) from 219 healthy controls and 87 diagnosed COVID-19 cases were detected and used to determine the cutoff value. As shown in **Figure 3A**, A p value of < 0.05 was considered statistically significant for all tests. AUC was 0.932 (95% CI: 0.894–0.970). The max Youden Index (Youden Index = Sensitivity + Specificity -1) was 0.846, with a cutoff of 0.0495, a sensitivity of 90.0%, and a specificity of 94.7%. Therefore, T/C 0.05 was set as cutoff value.

### Clinical Application and Evaluation

This part study was a randomized controlled and single-blind experiment. Clinical samples from 247 confirmed COVID-19 patients (male, n = 102, female, n = 145; age range 2–68, median 48), 443 patients with other respiratory diseases (male, n = 222, female, n = 221; age range 0–93, median 35) and 300 healthy people (male, n = 159, female=141; age range 20–58, median 32) were enrolled and detected by the antigen strips and the fluorescence PCR method, respectively. Finally, we evaluated the sensitivity and specificity of detection base on their clinical diagnosis after unblinding. Among the 247 COVID-19 patients, there were 137 patients (male, n = 47, female, n = 90; age range 2–86, median 49) at the disease progression stage (day 0–44 after onset of fever, average day 25), and 110 patients (male, n = 55, female, n = 55; age range 4–86, median 48) at the cured stage (days 19–55, average day 37). According to the discharge standard of COVID-19 by the National Health Committee, at the cured stage here means that the body temperature is normal for more than three days, the symptoms of respiratory tract are obviously improved, the CT image shows that the inflammation is obviously absorbed, and the nucleic acid test of respiratory tract pathogen is negative for two consecutive times. As shown in **Figures 3B–D** and **Table 5**, the results showed that the specificity of the test strip in healthy controls and patients with other respiratory disease was 100.00 and 97.29%, the sensitivity in cases at progress stage and cured stage was 67.15 and 7.02%. Compared to the results of RNA test, which is the most commonly used technology in clinic, the positive percentage agreement (PPA) and negative percentage agreement (NPA) of antigen strip were 83.16% (79/95) and 94.45% (562/595) (**Table 5**).

**Table 5:** The specificity and sensitivity of test strip.

Study group		No.test	Antigen Strip				RNA test			
			Positive	Nagetive	Sensitivity (%)	Specificity (%)	Positive	Nagetive	Sensitivity (%)	Specificity (%)
Healthy controls		300	0	300	–	100	–	–	–	–
Other respiratory disease		443	12	431	–	97.29	0	443	–	100.00
SARS-CoV-2 Samples		247	100	147	40.49	–	95	152	38.46	–
	Progress stage	137	92	45	67.15	–	95	42	69.34	–
	Cured stage	110	8	102	7.02	–	0	110	0	–
Total (without healthy controls)		690	112	578	–	–	95	595	–	–
No. agreement with RNA test		–	79	562	–	–	–	–	–	–
Percentage agreement with RNA test		–	83.2%*	94.5%**	–	–	–	–	–	–

*Positive Percentage Agreement, **Negative Percentage Agreement.

Among the 443 patients with other respiratory diseases, there were 68 influenza cases infected with Influenza A H1N1, Influenza A H3, Influenza B Victoria and Influenza B Yamagata. The strip has great identification ability, with a false positive rate of 1.5% (1/68). For the cases with COVID-19, we observed the antigen detection rate at different time of onset. As shown in **Figure 3C**, the detection rate of antigen was higher than 48% within 35 days after onset of fever. Its trend overall the development of disease was similar to that of RNA detection. The detection rates of both of the two methods were significantly reduced after 35 days, which were consistent with the course of disease.

If the antigen test was used to supple RNA detection for auxiliary diagnosis, the diagnostic value of combined evaluation increased to AUC of 0.865(95% CI: 0.822–0.909) (**Figure 3D**), while the diagnostic value of single antigen test and RNA test was 0.802(95% CI: 0.752–0.852) and 0.825(95% CI:0.776–0.874).

## Discussion

At present, the detection of SARS-CoV-2 mainly includes nucleic acid detection and immune detection. Nucleic acid testing, or molecular testing, is the gold standard for diagnosing infectious diseases by detecting the genetic material of viruses. However, this method is mainly performed by fluorescence quantitative PCR, which has the problems of complicated extraction process, high requirements of experimental environment and conditions, long detection time, and extremely high rate of missed detection. In addition, research has shown that the SARS-CoV-2 is a new type of RNA virus, and RNA is easily degraded during the extraction process (Landolt et al., 2016; Nwokeoji et al., 2016; Le Bleu et al., 2017), which will lead to the false negative results. Therefore, RNA cannot be adapted to the requirements of rapid test and a large number of suspected case screening. According to the experience obtained from the immunoassay of SARS virus, immunoassay can be used as a supplement to the nucleic acid detection of SARS-CoV-2, especially for suspected cases with similar clinical symptoms and negative nucleic acid test, which has a great complementary diagnostic effect (Li et al., 2003; Shi et al., 2003).

Immunoassay is based on the specific response between antigens and antibodies, by detecting viral proteins (antigens) in the body, or antibodies specific to viral proteins in the body to make a diagnosis. Immunoassay includes antigen detection and antibody detection. The antigen detection kit based on immunofluorescent microspheres only needs to add the sample to the lysate to lyse out the antigen in the sample for detection, which can avoid the tedium of quantitative PCR. Though the RNA extraction process greatly simplifies the experimental operation process and shortens the detection time, there still remain missing test. On the other hand, RNA detection is aimed at the detection of live virus, while antigen detection is aimed at virus protein. In principle, protein fragments can also be detected after virus rupture. Therefore, it is particularly important to perform antibody testing after infection as a complement to the deficiency.

By comparing several detection methods of the SARS-CoV-2, the detection time of nucleic acid is earlier, antigen detection is more convenient and faster, and the combined use can better meet the requirements of SARS-CoV-2 detection. In this study, the diagnostic value of combined evaluation increased to AUC of 0.865(95% CI: 0.822–0.909), while the diagnostic value of single antigen test and RNA test was 0.802(95% CI: 0.752–0.852) and 0.825 (95% CI: 0.776–0.874) (**Figure 3D**).

In order to improve the sensitivity of this method, high specific monoclonal antibodies with enough affinity were selected for the specific spatial structure (antigenic determinant) of the SARS-CoV-2 N protein. Then, the method of fluorescence immunolabeling combined with fluorescence photometric signal detection was chosen, which could improve the sensitivity of the analysis method by 1-2 orders of magnitude, when compared to the traditional colloidal gold particle labeling method. Data from multicenter clinical trials showed that the sensitivity of antigen strip in COVID-19 cases at progress stage was 67.15%, but the results were close to RNA test, with a PPA and NPA of 83.16 and 94.45%. On another hand, the detection rate of antigen strip in COVID-19 cases at cured stage 7.02%, except for the possibility of false positives, this result also indicated that antigen fragments may temporarily store in the body of the cured patients or antigen test may assist RNA detection as a discharge index. The product “New Coronavirus (SARS-CoV-2) N Protein Detection Kit (Fluorescence Immunochromatography)” have been certified by European Conformity (Mar. 13, 2020; certificate no. HKT-20200313-001) and obtained the Provisional Authorization issued by health science authority of Singapore (July 13, 2020; MDPA2020-98).

In the present study, the validation experiment for the activated SARS-CoV-2 virus was conducted in the P3 laboratory, and the clinical experiments were performed in the P2+ laboratory laboratories (medical staff using P3 protection). The test strips exhibited good performance in detecting the live virus. How about the detection of inactivated virus? The results before and after the inactivation were compared, and it was found that the protein destruction after inactivation would affect the detection results (data shown in another study). The average test result (T/C value) of 450 ng of purified N protein was 2.89 (positive) before inactivation, and 0.041 (negative) after inactivation (56°C treatment). Then, the N protein was mixed with the throat swab (obtained from healthy people) and nasal swab (obtained from healthy people), respectively. The detection results were significantly reduced after the sample inactivation. Although the activated virus was a limitation in the present study, this ensured the sensitivity and accuracy of the detection. Therefore, the test should be operated in the P2+ laboratory, which can much faster provide a preliminary result, and cooperate with the nucleic acid detection, thereby improving the efficiency and accuracy. On the other hand, the antigen test does not require trained specialists or large-scale equipment, making it possible to be easily used in healthy population screening, and the self-monitoring of patients after cure. We have done comparison tests in terms of simplicity and feasibility. An UV-light can be used as a detection instrument for the antigen strips with a high sensitivity. The detection results of UV-light (LoD 1 × 10^3^TCID_50_/ml) were consistent with that of Savant-100 Fluorescence immunochromatographic analyzer, which was used in the study (the data of detection and clinical trials were submitted to CE certification and Singapore HAS certification).These advantages would meet the need of “rapid, instrument-free antigen test” recommended by Mina and colleagues in a recent perspective paper (Mina et al., 2020). The commentary recommends that in addition to current RT-PCR fluorescent testing, lateral-flow testing methods that are rapid and instrument-free should be developed for use in school, airports, and even at home. Such rapid test methods would be able to diagnosis COVID-19 earlier before infected patients are able to transmit their infections to others. The investigators will continue to improve the method in further research, in order to increase the sensitivity and specificity of the strips for inactivated virus. Furthermore, the investigators hope to develop portable equipment, reagents and standards suitable for home use, similar to those used as a blood glucose meter.

## Data Availability Statement

The original contributions presented in the study are included in the article/supplementary materials. Further inquiries can be directed to the corresponding authors.

## Ethics Statement

The studies involving human participants were reviewed and approved by Ethics Committee of Chinese PLA General Hospital, No. 2020-001; Ethics Committee of Wuhan Huoshenshan Hospital, No. 2020-003; Ethics Committee of Chongqing Public Health Medical Center, No.2020-007-01. The ethics committee waived the requirement of written informed consent for participation.

## Author Contributions

KZ and KH designed the study. SL and KD established methods. LZ, YZ, LC, JH, and FL collected the clinical information and samples. YL, HL, JL, PS, YX, and JYW performed the detection. CZ and KH performed data analysis and wrote the manuscript. All authors contributed to the article and approved the submitted version.

## Funding

This work was supported by the Beijing Science and Technology Planning Project (no. Z201100005420022).

## Conflict of Interest

SL and YX were employed by the company Beijing Savant Biotechnology Co., Ltd.

The remaining authors declare that the research was conducted in the absence of any commercial or financial relationships that could be construed as a potential conflict of interest.
